# Transient receptor potential channel 1/4 reduces subarachnoid hemorrhage-induced early brain injury in rats via calcineurin-mediated NMDAR and NFAT dephosphorylation

**DOI:** 10.1038/srep33577

**Published:** 2016-09-19

**Authors:** Zhong Wang, Yibin Wang, Xiaodi Tian, Haitao Shen, Yang Dou, Haiying Li, Gang Chen

**Affiliations:** 1 Department of Neurosurgery & Brain and Nerve Research Laboratory, The First Affiliated Hospital of Soochow University, 188 Shizi Street, Suzhou 215006, China

## Abstract

Transient receptor potential channel 1/4 (TRPC1/4) are considered to be related to subarachnoid hemorrhage (SAH)-induced cerebral vasospasm. In this study, a SAH rat model was employed to study the roles of TRPC1/4 in the early brain injury (EBI) after SAH. Primary cultured hippocampal neurons were exposed to oxyhemoglobin to mimic SAH *in vitro*. The protein levels of TRPC1/4 increased and peaked at 5 days after SAH in rats. Inhibition of TRPC1/4 by SKF96365 aggravated SAH-induced EBI, such as cortical cell death (by TUNEL staining) and degenerating (by FJB staining). In addition, TRPC1/4 overexpression could increase calcineurin activity, while increased calcineurin activity could promote the dephosphorylation of N-methyl-D-aspartate receptor (NMDAR). Calcineurin antagonist FK506 could weaken the neuroprotection and the dephosphorylation of NMDAR induced by TRPC1/4 overexpression. Contrarily, calcineurin agonist chlorogenic acid inhibited SAH-induced EBI, even when siRNA intervention of TRPC1/4 was performed. Moreover, calcineurin also could lead to the nuclear transfer of nuclear factor of activated T cells (NFAT), which is a transcription factor promoting the expressions of TRPC1/4. TRPC1/4 could inhibit SAH-induced EBI by supressing the phosphorylation of NMDAR via calcineurin. TRPC1/4-induced calcineurin activation also could promote the nuclear transfer of NFAT, suggesting a positive feedback regulation of TRPC1/4 expressions.

About 10 per 100, 000 individuals were affacted by aneurysmal subarachnoid hemorrhage (SAH) each year^1^. Early brain injury (EBI) during the first 72 h after SAH is the most important determinant of clinical outcome^2^,^3^,^4^. Neuronal protective drugs were applied extensively in animal experiments and clinical researches^5^, but the exact molecular mechanism remains unclear yet^6^,^7^,^8^.

Transient receptor potential channel (TRPC) is a family of voltage-sensitive calcium ion channels. TRPC has a construction of six transmembrane, and TRPC has both N terminal and C terminal inside the membrane. Increasingly experimental researches in SAH have focused on EBI and the microcirculation. And TRPC family has been reported to be associated with cerebral vasospasm^9^. There are 4 subfamilies of TRPC, including TRPC1, TRPC2, TRPC3/6/7, and TRPC4/5^10^. Among them, the antibodies of TRPC1 and TRPC4 have been shown to inhibit the calcium influx induced by endothelin-1, and then the neuronal damage^11^.

N-methyl-D-aspartic receptor (NMDAR) is known to participate in brain injury after SAH^12^. The function of NMDAR is regulated by phosphorylation^13^. Calcineurin is highly expressed in the brain, and dephosphorylation by calcineurin is becoming a hot research topic in stroke^14^. Calcineurin might take part in the process of ischemic and hemorrhagic stroke via mediating the cofilin activation^15^. Nuclear factor of activated T cells (NFAT), a transcription factor promoting the expression of TRPC family, has been shown to express in neurogenic brain regions (such as hippocampus and subventricular zone) and in cultured neurons^16^. NFAT dephosphorylation induced by activated calcineurin is essential for the nuclear transfer and transcription factor activity of NFAT^17^. Recently, NFAT was also studied widely in central nervous system^18^,^19^.

In this research we aimed to find out the effects of TRPC1/4 on the outcomes in the rat SAH model and the underly mechanisms.

## Results

### The protein levels of TRPC1/4 were increased after experimental SAH

**Figure 1:**
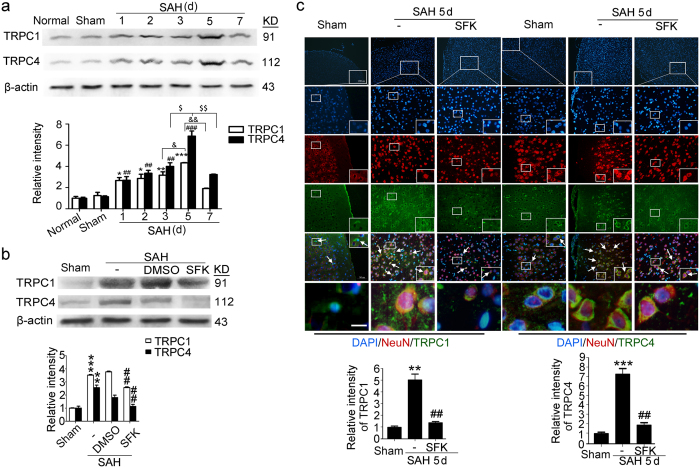
Effects of subarachnoid hemorrhage (SAH) stimulus and SFK96365 treatment on the protein levels of TRPC1/4. (**a**) Western blot assay of the effect of SAH stimulus. Data are means ± SEM. *p < 0.05, **p = 0.0054, ***p < 0.001, ^##^*p* < 0.01, ^###^*p* < 0.001 vs. sham group; ^&^*p* = 0.041, ^&&^*p* = 0.0067, ^$^*p* = 0.031, ^$$^*p* = 0.0064 (n = 6). (**b**) Western blot assay of the effect of SFK96365 treatment. Data are means ± SEM. **p = 0.006, ***p < 0.001 vs. sham group; ^##^*p* < 0.01 vs. SAH + DMSO group (n = 6). (**c**) Double-immunofluorescence. TRPC1 or TRPC4 (green) and neuronal marker (NeuN, red), and nuclei were fluorescently labeled with DAPI (blue). Arrows point to TRPC1-positive or TRPC4-positive neuronal cells. Scan bar = 32 μm. The relative fluorescent intensity of TRPC1 or TRPC4 in neuronal cells was shown. Data are means ± SEM. In TRPC1, **p = 0.003 vs. sham group, ^##^*p* = 0.004 vs. SAH group; In TRPC4,***p < 0.001 vs. sham group, ^##^*p* = 0.0051 vs. SAH group, n = 6.

Western blot results showed that the protein levels of TRPC1 and TRPC4 had a upward trend, and peaked at 5 d after SAH (Fig. 1a). To further study the potential roles of TRPC1/4 in SAH-induced EBI, TRPC1/4 inhibitor SKF96365 were used. Both western blot and immunofluorescent staining showed that the protein levels of TRPC1 and TRPC4 in brain, especially in neurons, were significantly decreased by SKF96365 treatment (Fig. 1b,c).

### Inhibition of TRPC1/4 by SKF96365 aggravated SAH-induced EBI

**Figure 2:**
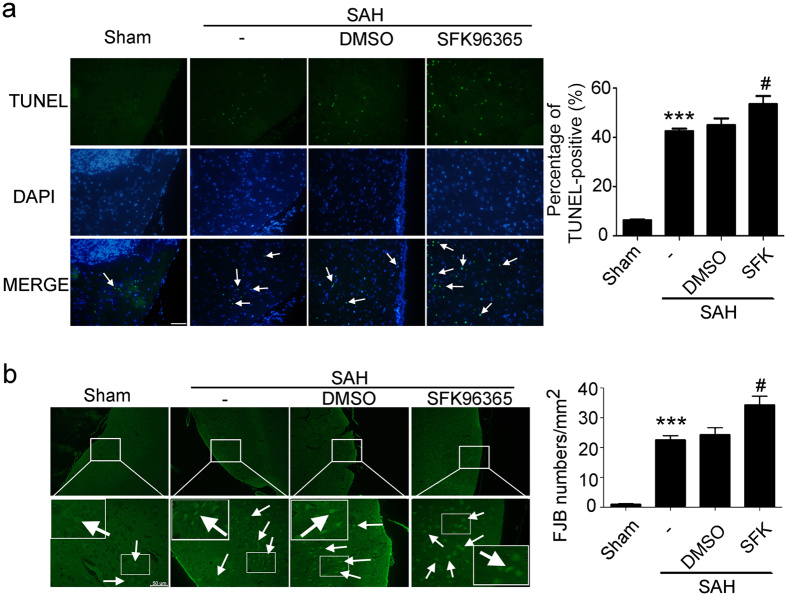
Effects of TRPC1/4 on early brain injury induced by subarachnoid hemorrhage (SAH). (**a**) Double staining of TUNEL (green) and DAPI (blue). Arrows point to TUNEL-positive cells in the brain. Scan bar = 100 μm. Data are means ± SEM. ***p < 0.001 vs. sham group, ^#^*p* = 0.049 vs. SAH + DMSO group (n = 6). (**b**) Fluoro-jade B (FJB) staining. Arrows point to fluoro-jade B-positive cells. Scan bar = 50 μm. Data are means ± SEM. ***p < 0.001 vs. sham group, ^#^*p* = 0.035 vs. SAH + DMSO group (n = 6).

Then, the effects of TRPC1/4 inhibitor SKF96365 on SAH-induced cell death and neuronal degeneration were tested. Terminal deoxynucleotidyl transferase-mediated dUTP nick end labeling (TUNEL) and fluoro-jade B (FJB) staining showed that SKF96365 treatment aggravated SAH-induced cell death and degeneration (Fig. 2a,b).

### *In vitro* rescue effects of TRPC1/4 on oxyHb-induced neuronal death

**Figure 3:**
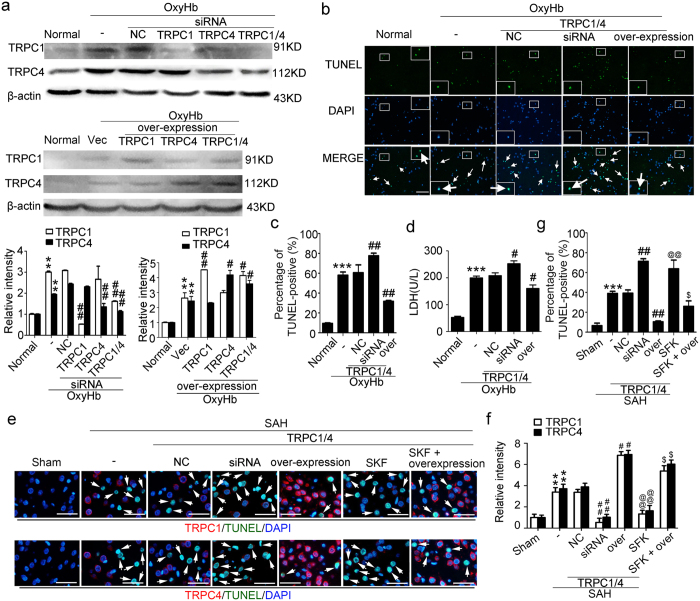
Effects of TRPC1/4 on cell death following subarachnoid hemorrhage (SAH). (**a**) Western blot analysis. Relative protein level was calculated based on densitometry analysis. Data are means ± SEM. **p < 0.01 vs. normal group; ^##^*p* < 0.01, ^#^*p* < 0.05 vs. oxyHb + negative control siRNA (NC) group or oxyHb + vector (vec) group (n = 3). (**b**,**c**) Double staining of TUNEL(green) and DAPI (blue) in cultured neurons. Arrows point to TUNEL-positive cells. Scan bar = 100 μm. Data are means ± SEM. ***p < 0.001 vs. normal group; ^##^*p* < 0.01 vs. oxyHb + negative control siRNA (NC) group (n = 3). (**d**) LDH activity in the culture medium. Data are means ± SEM. ***p < 0.001 vs. normal group; ^#^*p* < 0.05 vs. oxyHb + negative control siRNA (NC) group (n = 3). (**e**) Combination of TUNEL assay with immunofluorescence assay. Immunostaining of TRPC1 or TRPC4 (red) co-stained with TUNEL (green) in the brain at 72 h after SAH onsets. The nuclei were counterstained with DAPI (blue). Arrows point to TUNEL-positive cells co-labeled with low fluorescence intensity of TRPC1/4. Bar = 60 μm. The relative fluorescent intensity of TRPC1 or TRPC4 was shown in (**f**). Percentage of TUNEL-positive cells in the brain was shown in (**g**). Data are means ± SEM. ***p < 0.001, **p < 0.01, ^#^*p* < 0.05, ^##^*p* < 0.01 vs. SAH + negative control siRNA (NC) group, ^@@^*p* < 0.01 vs. SAH group, ^$^*p* < 0.05 vs. SAH + SFK 96365 group (n = 6).

OxyHb treatment significantly increased the protein levels of TRPC1/4 *in vitro*, which were significantly decreased by corresponding siRNA treatment and increased by expression plasmid treatment (Fig. 3a). TUNEL staining showed that neurons with upregulated TRPC1/4 had a lower death rate, while there was a higher death rate in neurons with siRNA interventions (Fig. 3b,c). Consistently, oxyHb-induced neuronal necrosis detected by LDH assay was enhanced by TRPC1/4 siRNA intervention and weakened by TRPC1/4 overexpression (Fig. 3d).

### *In vivo* rescue effects of TRPC1/4 on SAH-induced brain cell death

Considering the specificity of SKF96365, we also performed TRPC1/4 knockdown and overexpression in *in vivo* experiments. To further clarify the roles of TRPC1/4 in SAH-induced cell death, we co-stained brain sections for TRPC1/4 and TUNEL under indicated treatments (Fig. 3e). In line with the *in vitro* results, the protein levels of TRPC1/4 were significantly decreased by corresponding siRNA treatment and increased by expression plasmid treatment. And expression plasmid treatment reversed the SKF96365-induced decrease in the protein levels of TRPC1/4 (Fig. 3f). TUNEL staining showed that, compared with SAH + vector group, SAH + TRPC1/4 overexpression group had a lower death rate, while there was a higher death rate in SAH + TRPC1/4 siRNA group than SAH + negative control siRNA group. And TRPC1/4 overexpression significantly reversed the SKF96365-induced cell death, suggesting that SKF96365 should aggravate SAH-induced cell death via inhibiting TRPC1/4, at least partially (Fig. 3g). More interestingly, the co-staining results demonstrated that most TUNEL-positive cells were co-labeled with low fluorescence intensity of TRPC1/4, which further clarified the neuroprotection of TRPC1/4 against SAH-induced cell death (Fig. 3e).

### TRPC1/4 supressed the phosphorylation of NMDAR via calcineurin

**Figure 4:**
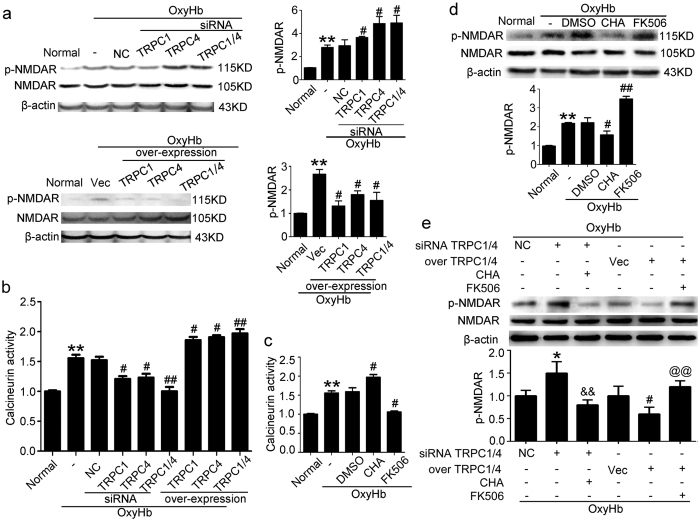
TRPC1/4 supressed the phosphorylation of NMDAR via calcineurin in neurons exposed to oxyHb. (**a**) Western blot analysis of the phosphorylation level of NMDAR in neurons. Data are means ± SEM. **p < 0.01 vs. normal group; ^#^*p* < 0.05 vs. oxyHb + negative control siRNA (NC) group or oxyHb + vector (Vec) group (n = 3). (**b**,**c**) Relative calcineurin activity. Data are means ± SEM. In (**b**), **p = 0.0084 vs. normal group; ^#^*p* < 0.05, ^##^*p* < 0.01 vs. oxyHb + negative control (NC) group (n = 3). In (**c**), **p = 0.0085 vs. normal group; ^#^*p* < 0.05 vs. oxyHb + DMSO group (n = 3). (**d**) Western blot analysis of the phosphorylation level of NMDAR in neurons. The mean values of normal groups were normalized to 1.0. Data are means ± SEM. **p = 0.0061 vs. normal group; ^#^*p* = 0.031, ^##^*p* = 0.0085 vs. oxyHb + DMSO group (n = 3). (**e**) Western blot analysis of the phosphorylation level of NMDAR in neurons. Data are means  ± SEM. *p = 0.039 vs. oxyHb + negative control siRNA (NC) group; ^&&^*p* = 0.0081 vs. oxyHb + TRPC1/4 siRNA group; ^#^*p* = 0.029 vs. oxyHb + vector (Vec) group; ^@@^*p* = 0.0069 vs. oxyHb + TRPC1/4 overexpression group (n = 3). CHA: chlorogenic acid.

OxyHb treatment significantly increased the phosphorylation level of NMDAR, which was enhanced by TRPC1/4 siRNA intervention and weakened by TRPC1/4 overexpression (Fig. 4a), suggesting that there were opposite trends between the phosphorylation level of NMDAR and the protein levels of TRPC1/4. Contrarily, the changes in calcineurin activity showed a consistent trend of TRPC1/4 protein levels (Fig. 4b). Calcineurin agonist chlorogenic acid (CHA) and calcineurin antagonist FK506 were used and the desired effects of them were verified. Under oxyHb treatment, calcineurin activity was significantly increased by CHA and decreased by FK506 (Fig. 4c). Moreover, CHA was shown to weaken oxyHb-induced the phosphorylation of NMDAR, while FK506 exerted an opposite effect (Fig. 4d). Finally, CHA was shown to restore the phosphorylation of NMDAR, even when siRNA intervention of TRPC1/4 was performed, while FK506 could weaken the dephosphorylation of NMDAR induced by TRPC1/4 overexpression (Fig. 4e). All the results suggested that TRPC1/4 could supress the phosphorylation of NMDAR via calcineurin.

### TRPC1/4 exerted neuroprotective effects in calcineurin dependent manner

**Figure 5:**
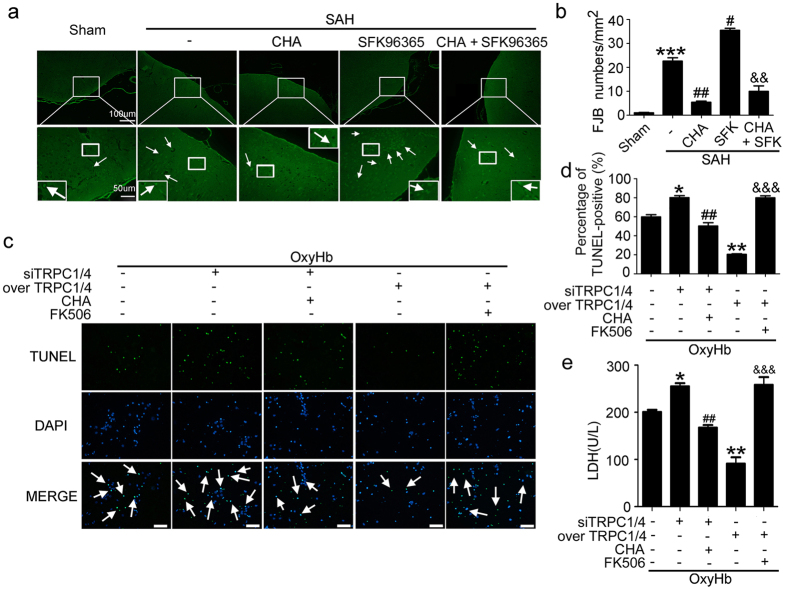
Roles of calcineurin in the neuroprotective effects of TRPC1/4 following subarachnoid hemorrhage (SAH). (**a**) Fluoro-jade B (FJB) staining. Arrows point to fluoro-jade B-positive cells. (**b**) Data are means ± SEM. ***p < 0.001 vs. sham group; ^##^*p* = 0.0043, ^#^*p* = 0.033 vs. SAH group; ^&&^*p* = 0.0051 vs. SAH + SFK group (n = 6). (**c**) Double staining of TUNEL (green) and DAPI (blue) in cultured neurons. Scan bar = 100 μm. Arrows point to TUNEL-positive cells. (**d**) Cell death rate. Data are means ± SEM. **p = 0.0051, *p = 0.047 vs. oxyHb group; ^##^*p* = 0.0071 vs. oxyHb + TRPC1/4 siRNA group; ^&&&^*p* < 0.001 vs. oxyHb + TRPC1/4 overexpression group (n = 3). (**e**) LDH activity in the culture medium. Data are means ± SEM. **p = 0.0068, *p = 0.048 vs. oxyHb group; ^##^*p* = 0.0091 vs. oxyHb + TRPC1/4 siRNA group; ^&&&^*p* < 0.001 vs. oxyHb + TRPC1/4 overexpression group (n = 3). CHA: chlorogenic acid.

FJB staining showed that CHA treatment could inhibit SAH-induced neuron degeneration and could effectively weaken SKF96365-induced deterioration in neuron degeneration under SAH condition (Fig. 5a,b). Consistently, the death (Fig. 5c,d) and necrotic (Fig. 5e) index of cultured neurons with oxyHb treatment showed the same trend: CHA treatment could effectively weaken TRPC1/4 siRNA intervention-induced deterioration in neuron death and necrosis, while FK506 could weaken the anti-death and anti-necrotic effects of TRPC1/4 overexpression.

### Calcineurin leaded to the nuclear transfer of NFAT

**Figure 6:**
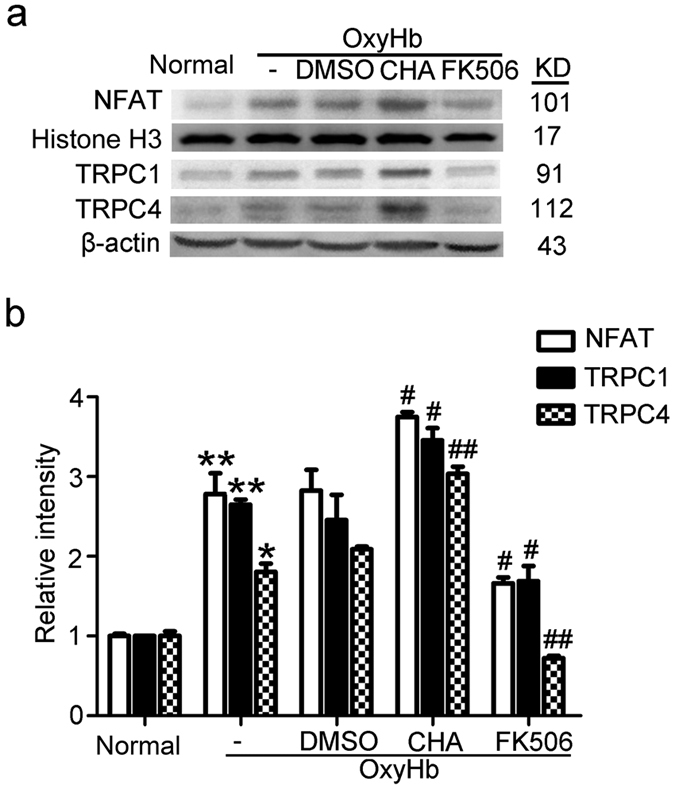
Roles of calcineurin in nuclear transfer of NFAT in neurons exposed to oxyHb. (**a**,**b**) Western blot analysis of the protein level of NFAT in nuclear protein and the protein levels of TRPC1/4 in total protein. Data are means ± SEM. **p < 0.01, *p = 0.027 vs. normal group; ^##^*p* < 0.01, ^#^*p* < 0.05 vs. oxyHb + DMSO group (n = 3). CHA: chlorogenic acid.

CHA treatment could significantly increase the nuclear transfer of NFAT accompanied by higher protein levels of TRPC1/4, while FK506 treatment exerted an opposite effect (Fig. 6a,b), which suggested that calcineurin promoted the nuclear transfer of NFAT, which might be a positive feedback regulation of TRPC1/4 expressions.

## Discussion

A large number of research focused on the expression and function of TRPC family. Among TRPC family, TRPC1 and TRPC4 have gained much attention in central nervous system. It has been reported that TRPC1 and TRPC4 participated in SAH-induced cerebral vasospasm^9^. TRPC1 and TRPC4 also have been shown to inhibit neuronal damage induced by endothelin-1^11^. And we found that TRPC1/4 could inhibit SAH-induced EBI via supressing the phosphorylation of NMDAR via calcineurin. Unconsistent effects of TRPC1 and TRPC4 on SAH-induced EBI and cerebral vasospasm make it cautious to choose intervention targeting TRPC1/4 for SAH treatment. In addition, cerebral vasospasm occurs and peaks within 4 to 9 days after the SAH^20^,^21^,^22^. So we assume that it is a double-edged sword that the expression of TRPC1/4 increased after SAH, which dominates the protective effect reducing the EBI, but the harmful effect remains further research while the expression of TRPC1/4 increased too high.

As TRPC1 and TRPC4 are from different subfamilies of TRPCs^10^ and there was more alteration of TRPC4 than that of TRPC1, it’s uncertain whether TRPC1 and TRPC4 have mutual influence on each other. There have been several studies focusing on the neuroprotective effects of TRPC. TRPC1 was reported to hold the ability to protect the neurons from the toxin-induced neuronal injury through opposite regulatory effects^12^. TRPC1 was also reported to have protective effect on human SH-SY5Y cells against cytotoxicity^23^. Furthermore, it was reported that the mammalian rods express two new calcium signalling mechanisms associated with SOCE and TRPC1 signalling might protect against prolonged calcium influx^24^. Yao *et al*.^25^ found the protective effect of TRPC against tat toxicity with CCL2. It was reported that TRPC channels were stimulated via the calcineurin/NFAT pathway signaling in the cardiomyocytes^26^. Many reseachs have been reported about the NMDAR, overactivation of NMDAR is a key event to cause the damage in central nervous system^24^,^27^. It’s reported that TRPC6 have protective effect in the ischemic model by inhibiting the NMDAR^28^,^29^. Our present study suggested that the TRPC1/4 inhibited the phosphorylation of NMDAR instead of its expression to protect neurons from excitotoxicity.

In this study, we applied SKF96365, FK506 and GHA to block TRPC1/4, inhibit CN and active CN, respectively. The specificity of these drugs should be discussed to present a more convincing link between TRPC and SAH-induced EBI in this study. Current researches have provided compelling evidences supporting the caution in the interpretation of results using SKF alone as a diagnostic agent for TRPC activity in native tissues. For example, SKF also exerts as a potent blocker of low-voltage-activated T-type calcium channels^30^, ATP sensitive K^+^ channels and voltage-gated K^+^ channels^31^. However, as described in several recent reports^32^,^33^,^34^, SKF96365 is still widely used as a pan TRPC inhibitor for the study of TRPC function both *in vivo* and *in vitro.* In addition, as shown in Fig. 1, SKF96365 indeed induced a decrease in the protein levels of TRPC1/4 in brain cells, especially neurons, in this SAH model. In addition, as shown in Fig. 3e,g, TRPC1/4 overexpression significantly reversed the SKF96365-induced cell death following SAH, suggesting that SKF96365 aggravated SAH-induced cell death via inhibiting TRPC1/4, at least partially. Beside CN, FK506 can also influence ryanodine receptor activity in vascular smooth muscle^35^. In this study, we focused on the role of CN in neurons following SAH. To avoided the potential side effects of FK506 in *in vivo* environment, we only used FK506 in *in vitro* experiments to treat cultured neurons. And as described in a recent report, FK506 was also used as a CN inhibitor to study the role of CN signaling in microcystin-LR triggered neuronal toxicity^36^. In addition, CHA has been proved to be an specific activator of CN^37^. And further pharmacological and toxicology experiment is still urgently needed to eliminate other potential side effects of them.

It has been reported that the positive feedback machenism of TRPCs was an important reason leading to the hypertrophy, while calcineurin-NFAT participated in the pathway^26^.

In addition, neurons together with astrocytes, microglia/macrophages, pericytes, brain microvascular endothelial cells and the extracellular matrix, constitute a “neurovascular unit”, which is essential for the function and health of CNS^38^. Since the neurovascular unit was first described by Zvi Cohen in 1996, the concept has been regarded as an elaborate web of microvessels in the brain, and has attracted a lot of attention in stroke researchers^39^,^40^. So, as the reviewer mentioned, the potential role of astrocytes and the vascular should be addressed. As shown in Fig. 3, both *in vitro* and *in vivo* rescue effects on oxyHb-induced neuronal death or SAH-induced brain cell death by TRPC1/4 overexpression are not complete. This failure may be due to the therapy solely targeting one cell type in isolation. Thus, protecting matrices and cells that support neurons, such as astrocytes, may be probably as important as protecting the neurons themselves. A key to overcoming the incomplete rescue of TRPC1/4 overexpression on SAH or induced cell death may be the new appreciation that aims the neurovascular unit as an integral part in the pathological process of SAH-induced EBI.

Finally, the contradictory role of TRPC1/4 after SAH, neuroprotective and promoting cerebral vasospasm, should be discussed. In this study, we found that TRPC1/4 could inhibit SAH-induced EBI by supressing the phosphorylation of NMDAR via calcineurin. However, TRPC1/4 also have been shown to promote the development of SAH-induced cerebral vasospasm^41^, which is an important factor involved in the mechanisms underlying the EBI after SAH^21^. For example, TRPC1 and TRPC4 participated in endothelin-1-induced Ca^2+^ influx in smooth muscle cells and then vasospasm after SAH^11^. TRPC1 could promote the vasospasm after SAH via mediating Ca^2+^ influx and phenotypic switching in smooth muscle cells^41^. Incubation with TRPC inhibitor SKF96365 or a function blocking TRPC1 antibody could delay the onset of vasomotion in the depolarisation initiating vasomotion model^42^. In conclusion, TRPC1/4 in smooth muscle cells could promote the development of SAH-induced cerebral vasospasm via regulating Ca^2+^ influx, while TRPC1/4 in neurons exerts a neuroprotective effect via CN/NMDAR pathway. Base on these study, new therapy specific up-regulating TRPC1/4 in neurons or specific down-regulating TRPC1/4 in smooth muscle cells will be more effective to prevent SAH-induced EBI.

There remained several limitations in this study. This experiment was performed on healthy adult rats, the results was difficult to speculate on SAH patients with basical vascular diseases. The effects of TRPC1/4 on NMDAR pathway, especially the subsequent excitability toxicity should be investigated further.

**Figure 7:**
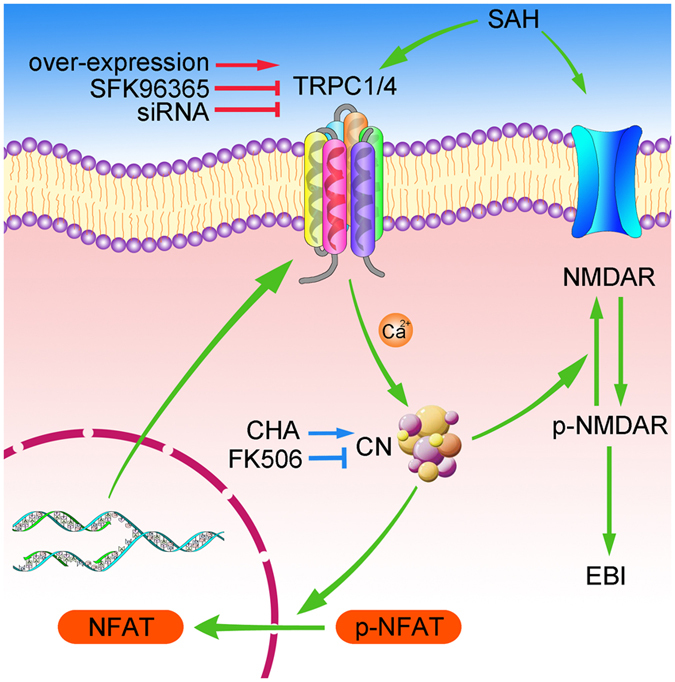
Mechanism of the protective effect induced by TRPC1 and TRPC4 after SAH. Following SAH, the expression of TRPC1/4 was increased, which in turn upregulated the activity of calcineurin (CN). CN promoted the dephosphorylation of NMDAR, which can restrict the early brain injury, including cortical death and degeneration due to SAH. With overexpression of TRPC1/4, more apparent protective effects were shown. With inhibition of TRPC1/4 (by inhibitor *in vivo* or by siRNA *in vitro*), EBI were aggravated. Activated CN promotes the nuclear transfer of NFAT, which in turn upregulates the expression of TRPC1/4. CHA: chlorogenic acid.

In conclusion, the main findings of this study are as follows (Fig. 7): 1) TRPC1/4 exert protective effects against SAH-induecd EBI, including cortical cell death and degenerating; 2) TRPC1/4 positively regulated the activity of calcineurin. 3) TRPC1/4 promoted the dephosphorylation of NMDAR via calcineurin, that may subsequently lead to a lowering excitatory amino acid toxicity and EBI; 4) NFAT dephosphorylation induced by activated calcineurin was essential for the nuclear transfer in this SAH model. To the best of our knowledge, this is the first study demonstrating such involvement.

## Materials and Methods

### Ethics statement

SD rats weighing between 350 and 400 g were provided by the Shanghai Experimental Animal Center of Chinese Academy of Sciences. All procedures were approved by the Institutional Animal Care Committee of Soochow University and were performed in accordance with the guidelines of the National Institutes of Health on the care and use of animals. All rats were placed under anesthesia before the fixation-perfusion and exsanguination euthanasia procedures.

### Rat SAH model

**Figure 8:**
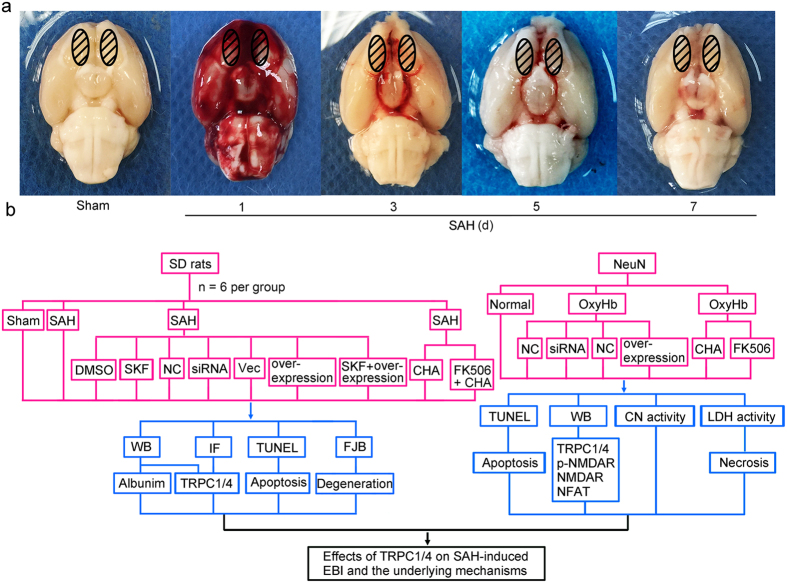
Experimental design. (**a**) The representative areas taken for assay. (**b**) Experiment design. NC: negative control siRNA; Vec: vector; CHA: chlorogenic acid.

Experimental SAH model was induced by single blood injection to prechiasmatic cistern in rats as reported previously^43^. In this model, the inferior basal temporal lobe of SAH group was stained with blood. Schematic representation of the areas taken for assay was shown in Fig. 8a.

### Cell cultures

Primary hippocampal neurons were harvested and cultured as described previously^43^,^44^.

### Study design

#### *In vivo* experiments

Firstly, 42 rats (50 rats were used, 42 rats were survived after the surgery) were randomly assigned to seven groups of 6 rats each, normal group, sham group and five experimental groups arranged by time: 1d, 2d, 3d, 5d and 7d after SAH (Fig. 8b). And then, 66 rats (83 rats were used, 66 rats were survived) were randomly divided into 6 groups: sham group, SAH group, SAH + DMSO group, SAH + SFK96365 group, SAH + negative control siRNA group, SAH + TRPC1/4 siRNA group, SAH + vector group, SAH + TRPC1/4 overexpression group, SAH + CHA group, SAH + FK506 group. The transfection of siRNA and plasmid in rat brain was performed 48 h before SAH onsets. And both CHA and FK506 were administered 30 min before induction of SAH and once a day until the rat was sacrificed. At 3d after SAH, which was chosen base on the results of the first experiment, and the brain cortex of 6 rats were extracted for TUNEL staining, FJB staining, immunohistochemical study, and western blot assay.

#### *In vivo* experiments

Firstly, primary hippocampal neurons were transfected with negative control siRNA, TRPC1 siRNA, TRPC4 siRNA, TRPC1 siRNA + TRPC4 siRNA, empty vector, TRPC1 plasmid, TRPC4 plasmid, TRPC1 plasmid + TRPC4 plasmid, respectively (Fig. 8b). Forty-eight hours after transfection, the cells were stimulated with 10 μmol/L OxyHb for an additional 24 h to mimic SAH condition as described previously^45^. The commercial oxyHb (Ruibio, O7109) was purchased and stored in low temperature under dim light to save the features of oxyHb. After the indicated treatments, cells were harvest for further analysis.

### Transfection of siRNA and plasmid in cultured neurons

Specific siRNAs and plasmid against TRPC1 and TRPC4 were obtained from GenScript. siRNA and plasmid transfection in cultured neurons was performed as described previously^43^. To improve the knockdown efficiency, the interference efficiency of 3 different siRNAs was test, and the most efficient one was used in the following study (data not shown).

### Transfection of siRNA and plasmid in rat brain

The transfection of siRNA and plasmid in rat brain was performed as described previously^43^. Briefly, according to the manufacturer’s instructions for Entranster-*in vivo* RNA transfection reagent (Engreen, 18668-11-1) and Entranster-*in vivo* DNA transfection reagent (Engreen, 18668-11-2), the Entranster-*in vivo*–siRNA mixture and Entranster-*in vivo*–plasmid mixture were prepared and injected intracerebroventricularly.

### Drug treatment

#### *In vivo* experiment

As a pan TRPC1/4 inhibitor, SKF96365 has been widely used^32^,^33^,^34^. However, in most of the reports, SKF96365 was used to treat detached tissue or cultured cells^32^,^33^,^34^. To explore s suitable dosage of SKF96365 for *in vivo* experiment, especially for this SAH model, we performed preliminary experiments. Briefly, TRPC1/4 inhibitor SKF96365 (B6616, Apexbio Technology LLC, US), prepared in DMSO at a concentration of 10 mM, was injected intraperitoneally at a dose range of 0.5–2.0 mg/kg body weight. In the experiment, we found that SKF96365 at 1.0 mg/kg body weight could effectively decrease the protein level of TRPC1/4 (data not shown). Then, we chose the dose of 1.0 mg/kg body weight in the following study. CN agonist chlorogenic acid (CHA, C3878, Sigma-Aldrich Co.LLC, US), prepared in DMSO at a concentration of 10 mM, was injected intraperitoneally at a dose of 1.0 mg/kg body weight^46^. *In vitro* experiment. CN antagonist FK506 (B2143, Apexbio Technology LLC, US) was prepared in DMSO at a concentration of 1 mM and was used to inhibit the activation of CN in culture neurons at a final concentration of 1 μM^36^. CN agonist CHA was prepared in DMSO at a concentration of 10 mM and was used to activate CN in culture neurons at a final concentration of 10 μM^47^.

### Antibodies

Rabbit anti-NMDAR1 monoclonal antibody (ab17345), rabbit anti-TRPC4 monoclonal antibody (ab84813), rabbit anti-NFAT2 monoclonal antibody (ab175134), and rabbit anti-Histone H_3_ antibody (ab8580) were from Abcam (Cambridge, MA, USA). Rabbit anti-TRPC1 antibody (sc-20110) and rabbit polyclonal anti-p-NMDAR1 antibody (sc-31669) were from Santa Cruz Biotechnology (Santa Cruz, CA, USA). Mouse anti-NeuN [1B7] antibody-Neuronal Marker (ab104224) was from Abcam. Normal rabbit IgG (sc-2027) and normal mouse IgG (sc-2025) were from Santa Cruz Biotechnology. Secondary antibodies for Western blot analysis, including goat anti-rabbit IgG-HRP (sc-2004), rabbit anti-goat IgG-HRP (sc-2768), and goat anti-mouse IgG-HRP (sc-2005), were from Santa Cruz Biotechnology. Secondary antibodies for immunofluorescence, including Alexa Fluor-488 donkey anti-rabbit IgG antibody (A21206), Alexa Fluor-488 donkey anti-goat IgG antibody (A11055), and Alexa Fluor-555 donkey anti-mouse IgG antibody (A31570), were from Invitrogen.

### Nuclear protein extraction

Nuclear protein extraction was performed using a Nuclear and Cytoplasmic Protein Extraction Kit from Beyotime (P0027).

### Calcineurin activity analysis

Calcineurin activity analysis was performed using a calcineurin activity quantification kit from GENMED SCIENTIFICS, INC. USA (GMS50042.1).

### Western blot analysis

The proteins from the frozen brain tissue or the cultured cells were extracted and analyzed by western blot as previously described^45^,^48^. The band signal was detected using enhanced chemiluminescence (ECL) kit (Beyotime, Shanghai, China). Finally, the relative quantity of proteins was analyzed using Image J and normalized to that of loading controls. Phosphorylation levels were evaluated by the ratio of phosphoprotein to total protein.

### TUNEL and FJB staining

Cell death was detected using TUNEL according to the manufacturer’s protocol (*In situ* cell death detecton kit,fluorescein, Roche, USA). FJB (Histo-Chem Inc., Jefferson, AR, US) was used as a marker of neuronal degradation and was perpormed as previously described^49^.

### Immunohistochemical study

Double immunofluorescence analysis was performed with antibodies for TRPC1 or TRPC4 and neuron markers (NeuN) as previously described^49^. Normal rabbit or mouse IgG was used as a negative control (data not shown).

### Statistical Analysis

Values are presented as means ± SEM. SPSS 11.5 (SPSS Inc., Chicago, IL, US) was used for statistical analysis. Statistical comparisons between groups were performed using one-way analysis of variance followed by either a Dunnett’s or a Tukey’s post hoc test, the former for comparisons to a single control group, the latter to compare across multiple groups. A probability of *P* < 0.05 was considered statistically significant.

## Additional Information

**How to cite this article**: Wang, Z. *et al*. Transient receptor potential channel 1/4 reduces subarachnoid hemorrhage-induced early brain injury in rats via calcineurin-mediated NMDAR and NFAT dephosphorylation. *Sci. Rep.* **6**, 33577; doi: 10.1038/srep33577 (2016).
